# Revision of the subgenus *Tinotus* Sharp, stat. n., of the parasitoid rove-beetle genus *Aleochara* Gravenhorst (Coleoptera, Staphylinidae, Aleocharinae) from Japan, Taiwan, and the Russian Far East

**DOI:** 10.3897/zookeys.559.6755

**Published:** 2016-02-03

**Authors:** Shûhei Yamamoto, Munetoshi Maruyama

**Affiliations:** 1 Entomological Laboratory, Graduate School of Bioresource and Bioenvironmental Sciences, Kyushu University, Hakozaki 6-10-1, Higashi-ku, Fukuoka 812-8581, Japan; 2 The Kyushu University Museum, Hakozaki 6-10-1, Higashi-ku, Fukuoka 812-8581, Japan; 3 Japan Society for the Promotion of Science Research Fellow (DC), Japan

**Keywords:** Aleocharini, new species, new combinations, revalidation, replacement names, checklist, East Asia, Palaearctic Region

## Abstract

The subgenus *Tinotus* Sharp, 1833, **stat. n.**, of the genus *Aleochara* Gravenhorst, 1802 (Aleocharini: Aleocharina) from Japan, Taiwan, and the Russian Far East is revised. *Tinotus* is a new record from the latter two regions. Three species are recognized: Aleochara (Tinotus) morion Gravenhorst, 1802, **comb. n.** [Japan (new record), the Russian Far East (new record)], Aleochara (Tinotus) eoa **nom. n.** [replacement name for *Tinotus japonicus* Cameron, 1933; Japan, Taiwan (new record)], and Aleochara (Tinotus) takashii **sp. n.** (central Honshû, Japan). The systematic position of *Tinotus* is discussed. All species are (re-)described, keyed, and figured. A world checklist of *Tinotus* species, comprising 40 valid species, is provided in an appendix. Additional taxonomic changes are proposed, including a new synonymy, a revalidation, 13 new replacement names, and 27 new combinations.

## Introduction

The rove-beetle genus *Aleochara* Gravenhorst, 1802 (Aleocharinae: Aleocharini, Aleocharina) is distributed worldwide, except in Antarctica (Klimaszewski 1984). This genus is the most speciose genus of the tribe Aleocharini, with approximately 500 species in 18 subgenera. *Aleochara* is likely a monophyletic group based on extensive molecular analyses (Maus et al. 2001).

On the other hand, the genus *Tinotus* Sharp, 1883 has recently been recognized as phylogenetically close to, or a possible member of, *Aleochara* (Maus et al. 2001; Hanley 2002; Osswald et al. 2013). Its taxonomic placement, including its tribal assignment, has been controversial for a long time, mainly due to its tarsal formula (4-5-5; see Hanley 2002). According to Maus et al. (2001), *Tinotus* is a taxon that should be considered within the genus *Aleochara*. The distributional range of *Tinotus* is quite wide, as it has been recorded from every zoogeographic region, except the Australian Region, with approximately 40 species (Hanley 2002; Klimaszewski et al. 2002).

Larvae of *Aleochara* and *Tinotus* act as ectoparasitoids on cyclorrhaphous Diptera, and the adults prey upon dipteran eggs and larvae (e.g., Klimaszewski 1984; Maus et al. 1998). Thus, they have been considered potential candidates for classic biological control of commercial crops against pest flies in Europe and North America (e.g., White and Legner 1966; Fournet et al. 2000).

Taxonomic knowledge of *Tinotus* in East Asia is still incomplete. In Japan, Taiwan, and the Russian Far East, just one species, *Tinotus japonicus* Cameron, 1933, has been originally described from Japan. In this study, we (re-)describe three *Tinotus* species distributed in these regions. We discuss the systematic position of *Tinotus*. We also provide a complete *Tinotus* species list, reflecting recent species additions, synonyms, and corrections.

## Material and methods

We used the technical procedures and methods used by Maruyama (2006) and Yamamoto and Maruyama (2012). The terminology used for this study generally follows that of Hanley (2002), Klimaszewski et al. (2002), and Yamamoto and Maruyama (2012, 2013). For chaetotaxy of the mouthparts we followed Sawada (1972, 1987). We followed Welch (1997) for genital terminology, especially that of females. In the descriptions, the number of macrosetae on tergite VIII and sternite VIII refers to one side of the body. Furthermore, minute setae were omitted or depicted only for one side of the body.

Abbreviations for measurements: BL, length of the body from clypeus to apex of the abdomen; EW, maximum width both elytra combined; HL, maximum length of the head; HW, maximum width of the head; PL, maximum length of the pronotum; PW, maximum width of the pronotum along midline.

Other abbreviations: BRL, blue round label pinned by a curator; HW, handwritten.

The following acronyms of museums and private collections are used throughout the text:

BMNH
Natural History Museum, London, U.K. (R. Booth) 


FMNH
Field Museum of Natural History, Chicago, U.S.A. (R. Baquiran) 


HUM
Hokkaido University Museum (M. Ôhara) 


KUM
Kyushu University Museum, Fukuoka, Japan (M. Maruyama) 


PCTW
 Private collection of Mr. Takashi Watanabe (Kanagawa, Japan) 


## Taxonomy

### Genus *Aleochara* Gravenhorst, 1802

#### Subgenus Tinotus

Taxon classificationAnimaliaColeopteraStaphylinidae

Sharp, 1883 stat. n.

Tinotus Sharp, 1883: 170. Type species: *Tinotus cavicollis* Sharp, 1883. Fixed by Fenyes 1918: 25, by subsequent designation.
Exaleochara Keys, 1907: 102. Type species: *Tinotus morion* Gravenhorst, 1802. Fixed by Klimaszewski et al. 2002: 284, by monotypy. As synonym of *Tinotus*: e.g., Bernhauer & Scheerpeltz 1926: 713; Blackwelder 1952: 163; Ashe 2000: 360; Hanley 2002: 457; Klimaszewski et al. 2002: 284; Gouix and Klimaszewski 2007: 30; Schülke and Smetana 2015: 505.
Acrimea Casey, 1911: 14. Type species: *Acrimea resecta* Casey, 1911. Fixed by Fenyes 1918: 20, by subsequent designation. Synonymized by Gusarov 2003: 353.
Tinotus See further references in Hanley (2002), Klimaszewski et al. (2002), and Gusarov (2003).

##### Diagnosis.

This subgenus is rather easily distinguished from the other congeneric taxa by 1) compact, small (< 4 mm), and 2) strongly spindle-shaped body; 3) 4-5-5 tarsal formula (5-5-5 in the other subgenera of *Aleochara*); 4) fully carinate mesoventrite; 5) wide and 6) truncate apex of intercoxal process of mesoventrite, 7) and its apex reaching to apex of intercoxal process of metaventrite; 8) median lobe of aedeagus with developed flagellum; 9) female spermatheca without apical invagination of spermathecal head (*sensu* Welch 1997) and, 10) coiled basally. Minute characters on mouthparts probably define the subgenus as well, e.g., setula *a* on the first segment of labial palpi located at nearly apical margin of the segment (Fig. 5; see also Sawada 1987).

##### Remarks.

See other characters mentioned in detail by Hanley (2002) and Klimaszewski et al. (2002).

##### Systematic position.

Sharp’s (1833) original description of *Tinotus* placed this taxon in the group Myrmedoniina (= Lomechusini) due to its 4-5-5 tarsal formula. Since, *Tinotus* has also been placed in Hoplandriini (e.g., Seevers 1978) or Aleocharini (e.g., Lohse 1974), mainly based on the presence of a pseudosegment on the maxillary and labial palpi (see Hanley 2002 for a historical review).

In contrast to these ambiguities, recent studies have refuted all tribal placements other than Aleocharini. Hanley (2002) recognized *Tinotus* within Aleocharini, suggesting a close relationship with the genus *Aleochara*, based on the seven shared morphological characteristics of the genus, e.g., bifid to crescent-shaped apex of the ligula. According to the extensive molecular study of *Aleochara* by Maus et al. (2001), *Tinotus* was fully resolved within the “*bilineata* clade” of *Aleochara*. Similarly, Osswald et al. (2013), who used significantly fewer species (only four species of *Aleochara*, one of which is *Tinotus*) but analyzed them with much more molecular markers (4599 bp), also supported the assignment of *Tinotus* to *Aleochara*.

In our morphological study of *Tinotus* and *Aleochara* species, we found numerous morphological similarities between these genera, including a long intercoxal process of mesoventrite, except for the 4-5-5 tarsal segmentation in *Tinotus* (5-5-5 in *Aleochara*). Among the subgenera of *Aleochara*, Tinotus shares characters with the subgenus Xenochara Mulsant & Rey, 1874, i.e., carinate mesoventrite and fusiform body (including convexed pronotum). Remarkably, the subgenus *Coprochara* Mulsant & Rey, 1874 seems to be significantly more closely related to *Tinotus*. In fact, they share some important characters, including a completely carinate mesoventrite and a coiled spermatheca (Yamamoto and Maruyama 2013). Maus et al. (2001) also implied that both subgenera are phylogenetically close to *Tinotus*. Reduction of the antennal segment or tarsal segmentation in Aleocharinae is associated rather frequently with miniaturization of their body size (e.g., tribes Hypocyphtini and Mesoporini), and *Tinotus* species are possibly no exception. Therefore, no significant character exists to distinguish *Tinotus* from *Aleochara* at the genus level. We herein transfer *Tinotus*, as the 19th subgenus, to the genus *Aleochara*.

#### Aleochara (Tinotus) morion

Taxon classificationAnimaliaColeopteraStaphylinidae

Gravenhorst, 1802 comb. n.

Figs 1 , 4–8 , 9–11 , 12–19 , 36

Aleochara morion Gravenhorst, 1802: 97 (original description).
Tinotus morion : Seevers 1978: 196 (male genitalia figured); Hanley 2002: 463 (catalogue of world species of *Tinotus*); Klimaszewski et al. 2002: 285 (key to Nearctic species of *Tinotus*), 294 (redescription); Smetana 2004: 362 (catalogue of Palearctic species of Aleocharinae); Gouix and Klimaszewski 2007: 30 (catalogue of Canadian and Alaskan species of Aleocharinae), 149 (dorsal habitus photographed); Klimaszewski et al. 2013: 16 (catalogue of Canadian Staphylinidae), 60 (redescription), 247 (dorsal habitus photographed), 273 (male and female genitalia figured); Schülke and Smetana 2015: 506 (catalogue of Palearctic species of Aleocharinae).
Aleochara (Tinotus) morion See other references and synonymies in Hanley (2002) and Klimaszewski et al. (2002).

##### Type locality.

Braunschweig, Germany.

##### Non-type material examined.

**JAPAN: Hokkaidô**: 1 male, Nemuroshibetsu, Shibetsu-chô, 18.vii.1977, S.-I. Naomi leg. (KUM); 1 male, Lake Toro, Shibecha, 27.vii.1986, S. Nomura leg. (KUM); 1 male, 3 spec., Kamishumbetsu, Betsukai-chô, 20.vii.1977, S.-I. Naomi leg. (KUM); 1 male, Mt. Mashû-dake (just below the summit), 820 m, Teshikaga-chô, 15.vii.1990, sweeping of *Carex*-grass, K. Haga leg. (KUM); 1 female, Shiretoko-tôge Pass, Rausu-chô, 3.viii.1989, bottom of gutter on roadside, K. Haga leg. (KUM); 1 male, 1 spec., Sakae-machi, Oshidomari, Rishirifuji-chô, S.-I. Naomi leg. (KUM); 2 females, 1 spec., Nukanan Dam (right bank), Memuro, Ashoro-chô, 30.vii.1988, human excrement, K. Haga leg. (KUM); 1 male, 1 female, 7 spec., Shihoro, Kamishihoro-chô, GPS 43°32’03.9”N, 143°09’58.5”E, 13.vii.2014, bear dung, S. Yamamoto leg. (KUM); 2 males, 1 female, 1 spec., Obihiro-shi, 6.vi.1980, H. Togawa leg. (KUM); 2 spec., Obihiro-shi, 7.vii.1980, H. Togawa leg. (KUM); **Honshû**: 1 female, Inashiki, Ibaraki-ken, 29.iv.1983, S. Ohmomo leg. (KUM); 1 male, Sugaya, Ranzan-machi, Saitama-ken, 10.iv.1994, K. Toyoda leg. (KUM); 1 female, Mt. Gagyû-san, Takahashi-shi, Okayama-ken, 29.v.1977, S.-I. Naomi leg. (KUM). **RUSSIA: Far East**: 1 male, Maltsevskaya Cape, Churkin, Vladivostok, Primorsky, 22.vi.1997, human excrement, M. Ôhara leg. (HUM).

##### Reference material examined.

**AUSTRIA: Niederösterreich**: 1 spec., “Ulrichskirchen / N. Ö., J. Spurny // morion [HW] / grh. [HW] // Chicago NHMus / M. Bernhauer / Collection” (FMNH); **ITALY: Calabria**: 1 spec., “Calabria / Cimina / lg. Paganetti // morion [HW] / grh. [HW] / det. Bernh. // Chicago NHMus / M. Bernhauer / Collection” (FMNH).

##### Diagnosis

**Figures 1–3.**
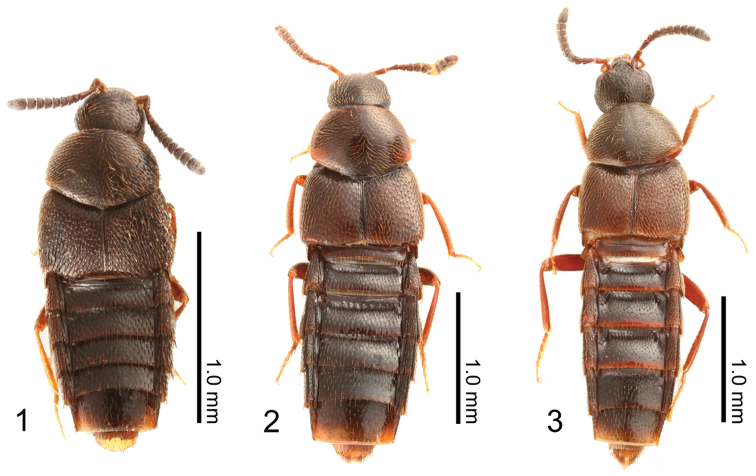
Dorsal habitus of Japanese species of Aleochara (Tinotus): **1** Aleochara (Tinotus) morion **2** Aleochara (Tinotus) eoa **3** Aleochara (Tinotus) takashii.

(see Klimaszewski et al. 2002). This species can be distinguished from most members of the subgenus *Tinotus* by the following combination of characters: body entirely black, rarely light brown (Fig. 1); median lobe of aedeagus of male with a basal protuberance in lateral view (Fig. 16: arrow); spermatheca with a simple and oblong spermathecal head, equally serrate inner walls inside spermathecal head, and with four coils at base (Fig. 19). *Aleochara morion* is extremely similar to Aleochara (Tinotus) rougemonti (Pace, 1993), comb. n. from China, including male-female genital structures, but the former species is discriminated from the latter by having longer sclerites inside a median lobe of the male aedeagus (Pace 1993: Fig. 160), and by having four coils of the female spermatheca (*Aleochara rougemonti* with two coils; Pace 1993: fig. 162).

##### Redescription.

*Measurements* (in mm, n = 30): BL = 2.448 (1.777–2.996); HL = 0.382 (0.315–0.453); HW = 0.392 (0.332–0.451); PL = 0.421 (0.355–0.485); PW = 0.632 (0.518–0.724); EW = 0.737 (0.595–0.853).

*Body* (Fig. 1): fusiform, compact, and robust; dorsal surface moderately glossy and pubescent, covered with large micro-reticulation. Color (Fig. 1): usually uniformly black to blackish brown; antennomeres I–III dark brown, but segments IV to X darker with numerous minute whitish setae; mouthparts and legs yellowish brown to brown; pubescence yellowish brown to brown.

*Head* (Fig. 1): subquadrate, as long as width (HW/HL = 1.03, n = 30), widest at just behind eyes; setae on vertex rather dense, directed anteriomedially. Eyes: small, occupying approximately one third of head length, very slightly protruding laterally.

*Mouthparts* (Figs 5–8): labrum (Fig. 7) moderately transverse, approximately 1.70 times as wide as long (excluding basal apodeme), anterior margin slightly emarginate medially, basal apodeme semi-transparent; surface with pseudopores scattered scarcely. Labial palpus (Fig. 5): setula *a* well-developed, situated near apical margin of labial palpomere I, while that of *b* and *c* strongly reduced. Maxilla (Fig. 6): lacinia with a distal comb consisting of dense-thin spines; galea rather short, as long as maxillary palpomere II; maxillary palpomere IV narrow and short, less than half length of that of III. Mentum (Fig. 8): anterior margin broadly emarginate.

**Figures 4–8.**
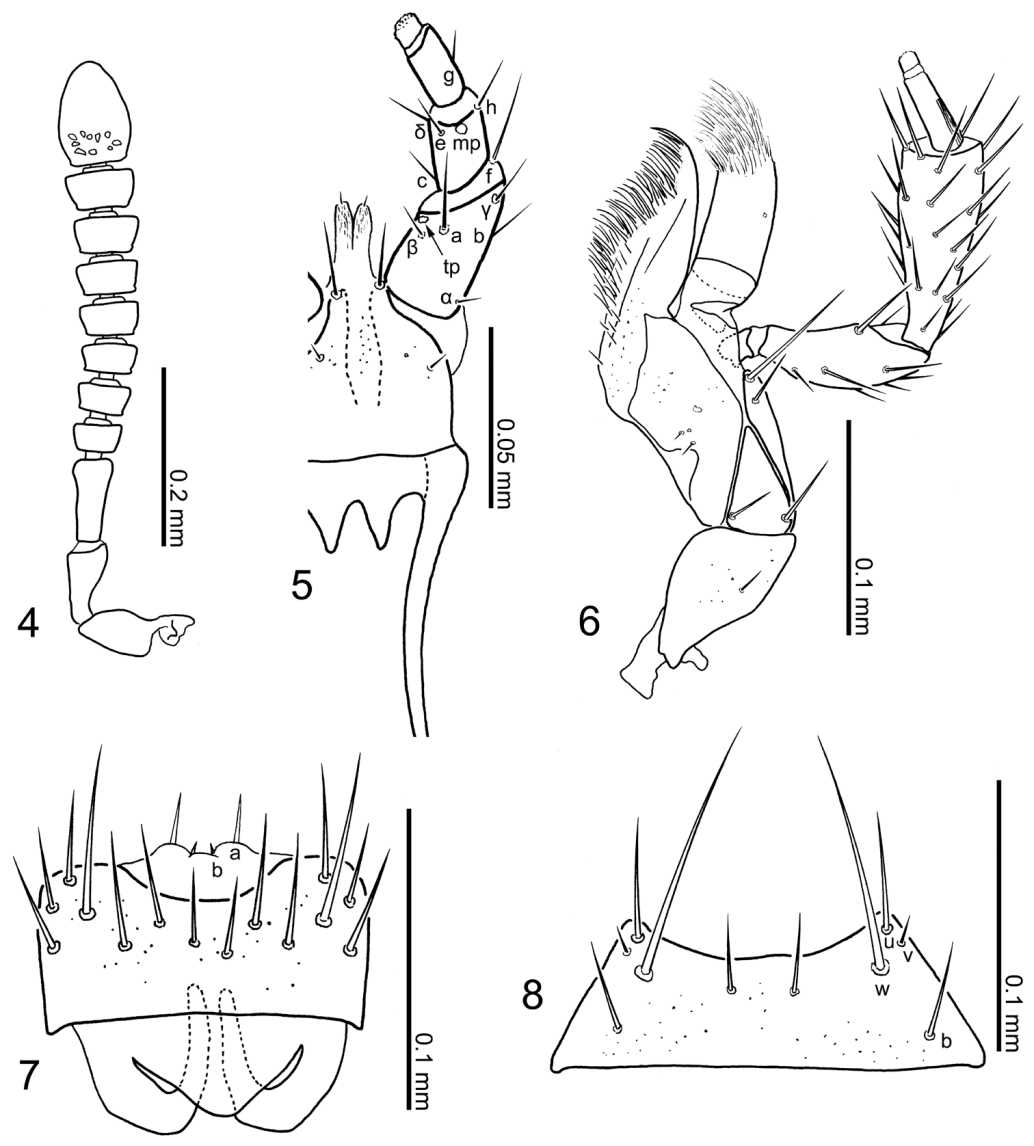
Body parts of Aleochara (Tinotus) morion of male: **4** right antenna **5** labium **6** maxilla **7** labrum **8** mentum.

*Antennae* (Fig. 4): short, moderately shorter than head and pronotum combined; thick, setaceous, becoming gradually and slightly broaden apically in segments IV to X, with segments IV to X clearly transverse; segment XI symmetrical, obtusely pointed at apex; approximate relative length of segments from basal to apex: 22: 14: 16: 5: 5: 5: 6: 6: 7: 7: 19.

*Pronotum* (Fig. 11): convex above dorsally, transverse (PW/PL = 1.50, n = 30), moderately longer than sutural length of elytra, widest around below of basal half, basal margin weakly rounded; pubescence rather long, dense, directed laterally and posterolaterally; micro-reticulation conspicuous.

**Figures 9–11.**
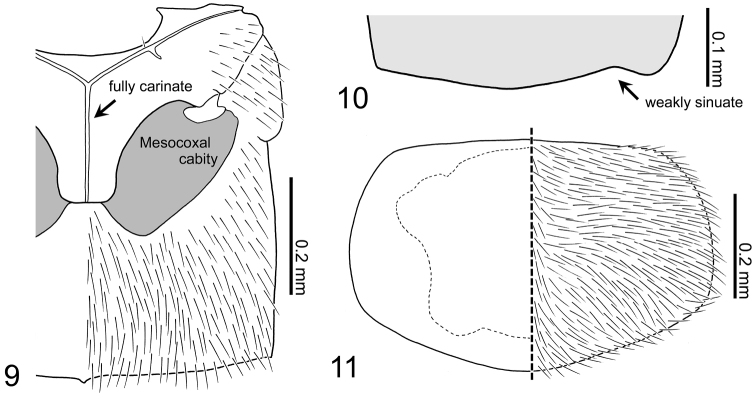
Body parts of Aleochara (Tinotus) morion of male: **9** mesoventrite and metaventrite **10** right elytron, posterior margin **11** pronotum.

*Mesoventrite* (Fig. 9): completely carinate along midline; inter coxal process broadly elongate, with truncate apex, completely reaching to inter coxal process of metaventrite.

*Elytra* (Figs 1, 10): together, transverse, rather small, widest at middle; pubescence short, finely scattered densely, diverging posterolaterally in each elytron; dorsal surface rough, somewhat deeply impressed; posterolateral corner of each elytron moderately sinuate.

*Legs* (Fig. 1): simple, short and, moderately slender; fore and midtibia with dozens of undeveloped spines, respectively.

*Abdomen* (Fig. 1): first three visible tergites rather shallowly impressed transversely at base; dorsal and ventral surface covered with setae densely.

**Figures 12–19.**
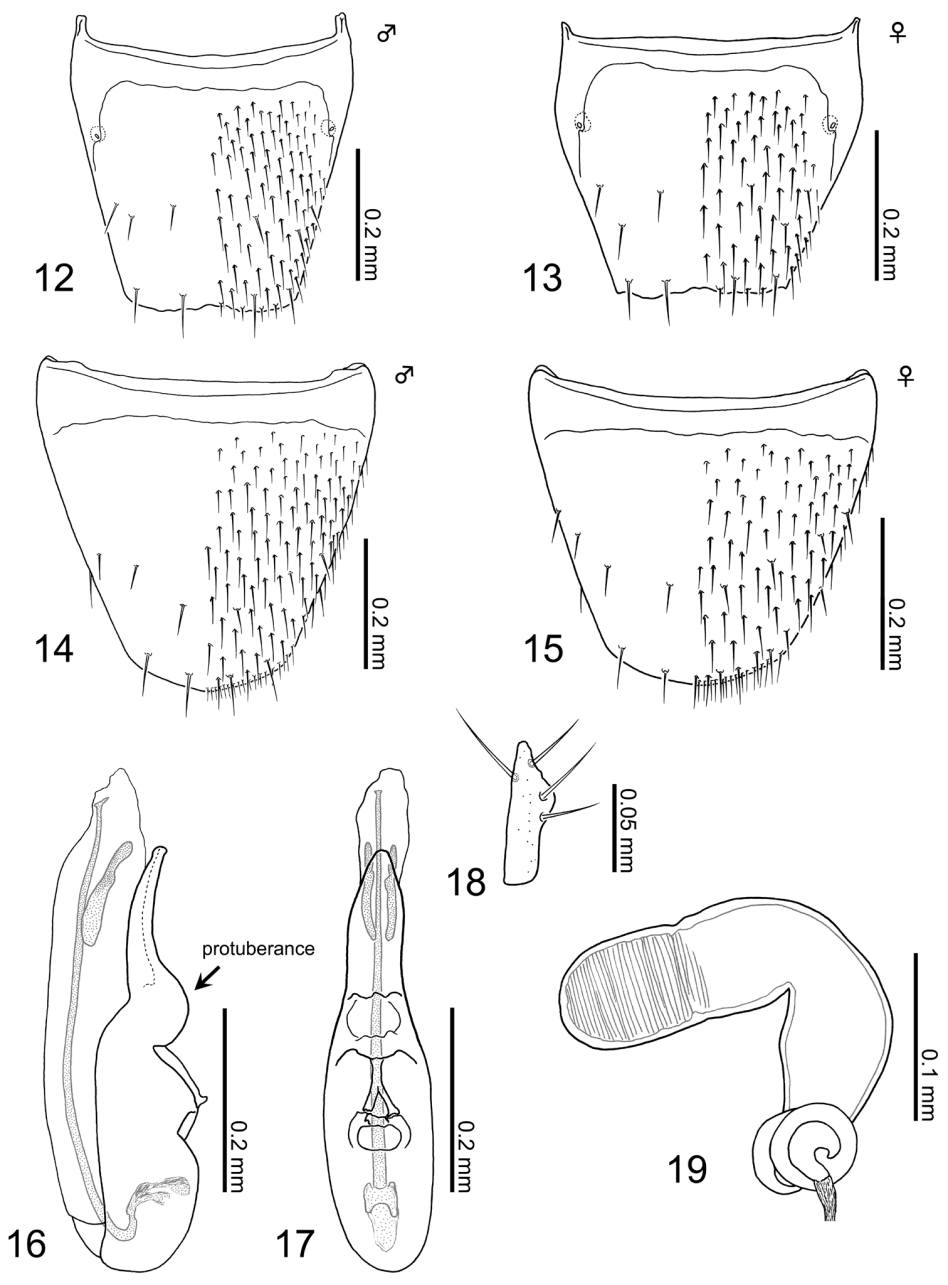
Terminalia of Aleochara (Tinotus) morion: **12** tergite VIII of male **13** tergite VIII of female **14** sternite VIII of male **15** sternite VIII of female **16** median lobe of male aedeagus, lateral view **17** ditto, parameral view **18** apical lobe of paramerite **19** female spermatheca.

*Male*. Tergite VIII (Fig. 12): basal suture fully developed (see Maruyama 2006: 20); posterior margin very weakly serrate, insignificantly emarginate medially; dorsal surface covered with setae rather densely, with five macrosetae. Sternite VIII (Fig. 14): basal suture fully developed; posterior margin very weakly pointed; ventral surface covered with setae densely, with approximately six macrosetae. Median lobe of aedeagus (Figs 16, 17): very slender in parameral view; apical lobe slender, weakly narrowing apically, and gently curved paramerally in lateral view; a conspicuous protuberance present at base of apical lobe (see arrow); a pair of simple sclerites, narrowly elongate, longer than half length of apical lobe; flagellum well developed, slightly shorter than median lobe, sharply curved near basal plate. Apical lobe of paramerite (Fig. 18): narrowly elongate, widest just above middle, with sharply pointed apex.

*Female*. Tergite VIII (Fig. 13): basal suture fully developed; posterior margin very weakly serrate, insignificantly emarginate medially; dorsal surface covered with setae rather sparsely, with five macrosetae. Sternite VIII (Fig. 15): basal suture fully developed; posterior margin rounded; ventral surface covered with setae densely, with approximately seven macrosetae. Spermatheca (Fig. 19): L-shaped; spermathecal head and neck fused together, forming a narrowly elongate capsule; attachment of spermathecal duct inconspicuous; basal part of spermathecal stem moderate in size, slightly longer than spermathecal neck, with four coils attached at base; each part of spermatheca entirely and very weakly sclerotized; inner wall of spermathecal head and neck, along border with head, finely and densely striate.

##### Distribution.

This species has a wide range in distribution covering the entire Holarctic region, mainly Europe and North Africa (Schülke and Smetana 2015). The records in the Nearctic region are regarded as those species introduced from Europe (Klimaszewski et al. 2002). In Russia, the easternmost record reported is from West Siberia (Schülke and Smetana 2015). We provide new distributional records of *Aleochara morion* as follows: new country record, Japan (Hokkaidô, Honshû); new regional record, the Russian Far East.

##### Bionomics.

SY collected eight specimens from one Hokkaido brown bear (*Ursus arctos*) dung found on the roadside of a mixed needleleaf and broadleaf forest in Hokkaidô, Japan (Fig. 36). *Aleochara morion* has been found among various habitats, such as decaying organic matter, including fungi, compost, animal excrement, and carrion (Horion 1967). In addition, this species is found in moss, bark debris, straw, hay, and on sandy soil (Klimaszewski et al. 2002).

##### Host records.

Three dipteran families are known as its host (Maus et al. 1998): Sepsidae, Drosophilidae, and Sarcophagidae.

##### Remarks.

Whether this species is native to East Asia or just an introduction from Europe is unknown, although the records from North America suggest this species has been introduced (Klimaszewski et al. 2002). Recently, Pace (2013) recorded *Aleochara morion* on the mainland of China.

#### Aleochara (Tinotus) eoa nom. n.

Taxon classificationAnimaliaColeopteraStaphylinidae

Figs 2 , 20–26 , 35

Tinotus japonicus Cameron, 1933: 217 (original description).
Tinotus japonicus : Smetana 2004: 362 (catalogue of Palearctic species of Aleocharinae); Shibata et al. 2013: 106 (catalogue of Japanese species of Staphylinidae); Schülke and Smetana 2015: 505 (catalogue of Palearctic species of Aleocharinae).

##### Type locality.

Kobe, Japan.

##### Type material examined.

*Tinotus japonicus*: Lectotype (here designated): male, “SYN- / TYPE [BRL] // JAPAN / Kobe // J. E. A. Lewis // M. Cameron / Bequest. / B. M. 1955-147 // Tinotus / japonicus / TYPE Cam [HW] // Tinotus / japonicus / P. M. Hammond / det. 1973 / SYNTYPE // Lectotype / Tinotus japonicus / Cameron, 1933 / des. Maruyama, 2011” (abdominal segments VIII-X and aedeagus were dissected and mounted in Euparal by MM) (PL, 0.42 mm; PW, 0.59 mm; Hind tibial length, 0.40 mm) (BMNH). Paralectoypes: 3 males, 1 female, same original labels as lectotype but without the label “Tinotus / japonicus / TYPE Cam [HW]” (abdominal segments VIII-X and spermatheca were dissected and glued on paper card together with body by MM) (BMNH).

##### Additional material examined.

**JAPAN: Honshû**: 1 female, Shigasaka-tôge Pass, Kanna-machi, Gunma-ken, 17-19.vi.2008, Flight Interception Trap, T. Watanabe leg. (KUM); 1 male, 2 females, Sugaya, Ranzan-machi, Saitama-ken, 10.iv.1994, K. Toyoda leg. (KUM). **TAIWAN: Nantou**: 1 male, 3 females, 5 spec., Songkang, 2000m, 14.iv.1986, M. Ôhara leg. (KUM).

##### Diagnosis.

This species is distinguished from the other congeneric species of the subgenus by a following combination of character states: body reddish brown to dark brown (Fig. 2); median lobe of aedeagus of male with a coiled flagellum, and with two pairs of characteristic sclerites (Figs 24, 25); spermatheca with a curved spermathecal head, unequally serrated inner walls inside spermathecal head, and with multiple coils at base (Fig. 26). *Aleochara eoa* is the most similar externally to Aleochara (Tinotus) rougemontiana (Pace, 1999a), comb. n., from mainland China, differing from it additionally by having much less coiled spermatheca in the female (Pace 1999a: Fig. 183).

**Figures 20–26.**
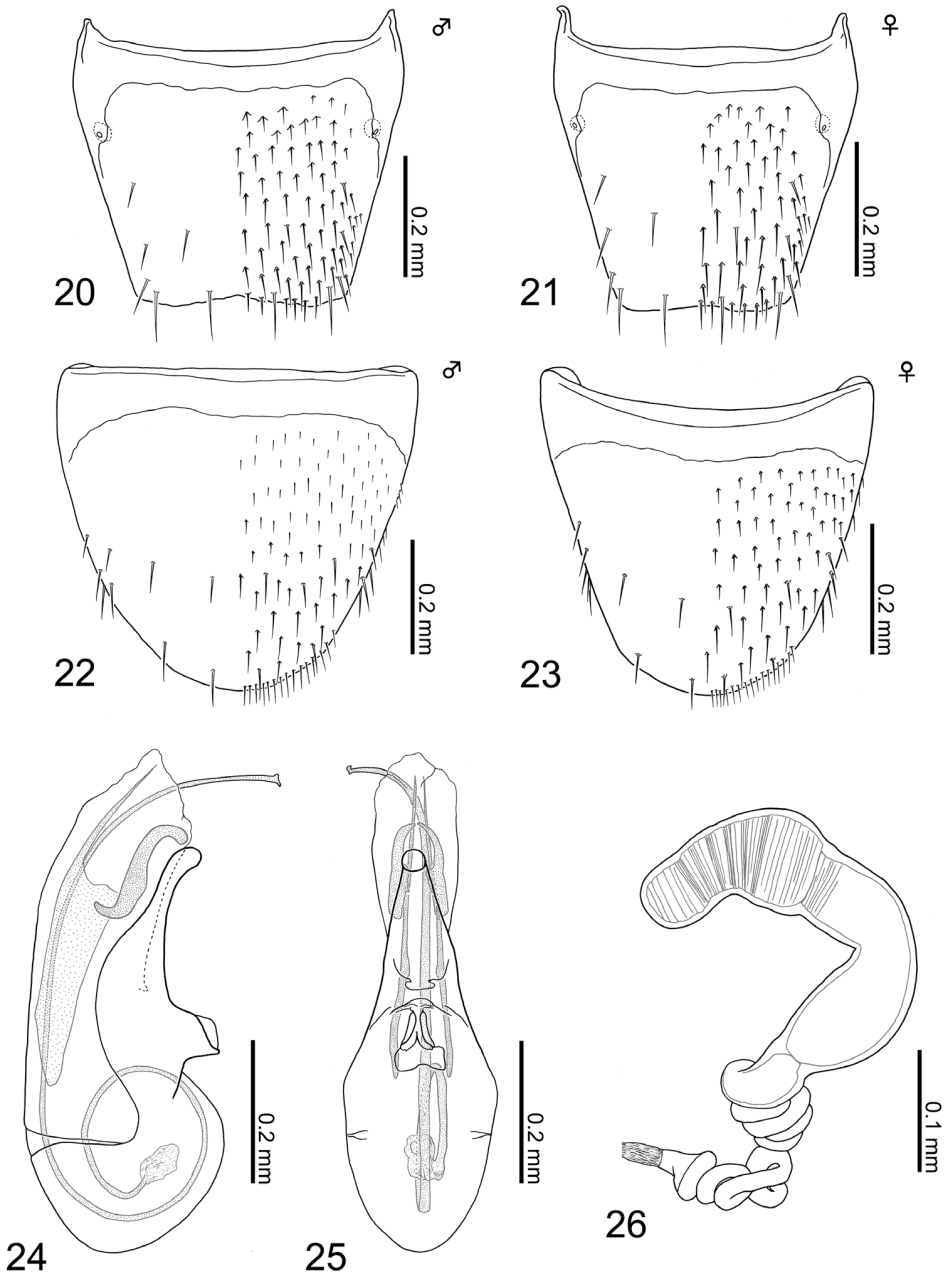
Terminalia of Aleochara (Tinotus) eoa: **20** tergite VIII of male **21** tergite VIII of female **22** sternite VIII of male **23** sternite VIII of female **24** median lobe of male aedeagus, lateral view **25** ditto, parameral view **26** female spermatheca.

##### Redescription.

*Measurements* (in mm, n = 13): BL = 2.709 (2.288–3.011); HL = 0.427 (0.358–0.511); HW = 0.439 (0.380–0.486); PL = 0.466 (0.368–0.565); PW = 0.666 (0.514–0.758); EW = 0.780 (0.605–0.948).

*Body* (Fig. 2): fusiform, compact, and robust; dorsal surface somewhat strongly glossy and pubescent, covered with small and inconspicuous micro-reticulation.

*Color* (Fig. 2): usually uniformly dark reddish brown to dark brown; antennomeres I–IV much lighter, but segments V to XI darker with numerous minute whitish setae; mouthparts and legs light-yellowish brown to reddish brown; pubescence yellowish brown to brown.

*Head* (Fig. 2): subquadrate, as long as width (HW/HL = 1.03, n = 13), widest at base of eyes; setae on vertex rather dense, directed anteriomedially. Eyes: small, occupying approximately one third of head length, very slightly protruding laterally.

*Antennae* (Fig. 2): short, moderately shorter than head and pronotum combined; relatively thick, setaceous, becoming gradually and slightly broaden apically in segments IV to X, with segment V spherical and segments VI to X clearly transverse; segment XI symmetrical, obtusely pointed at apex; approximate relative length of segments from basal to apex: 21: 17: 14: 6: 7: 7: 7: 7: 7: 7: 18.

*Pronotum* (Fig. 2): strongly convex above dorsally, transverse (PW/PL = 1.43, n = 13), moderately longer than sutural length of elytra, widest around below of basal half, basal margin weakly rounded; pubescence rather long, rather dense but thin, directed laterally and posterolaterally; micro-reticulation inconspicuous.

*Elytra* (Fig. 2): together, transverse, rather small, widest at middle; pubescence short, finely scattered densely, diverging posterolaterally in each elytron; dorsal surface moderately rough, shallowly impressed; posterolateral corner of each elytron moderately sinuate.

*Abdomen* (Fig. 2): first three visible tergites rather shallowly impressed transversely at base; dorsal and ventral surface covered with setae densely.

*Male*. Tergite VIII (Fig. 20): basal suture fully developed; posterior margin very weakly serrate, insignificantly emarginate medially; dorsal surface covered with setae rather sparsely, with six macrosetae. Sternite VIII (Fig. 21): basal suture fully developed; posterior margin rounded to only weakly produced; ventral surface covered with short setae sparsely, with approximately nine macrosetae. Median lobe of aedeagus (Figs 24 & 25): ovular in lateral and limuloid in parameral view; apical lobe rather slender, gently curved paramerally, weakly narrowing apically in parameral but with weakly dilated apex in lateral view; without a protuberance at base of apical lobe; a pair of sclerites S-shaped, longer than half length of apical lobe; flagellum strongly developed, much longer than median lobe, coiled 1.5 times at base.

*Female*. Tergite VIII (Fig. 21): basal suture fully developed; posterior margin very weakly serrate or almost truncate; dorsal surface covered with setae rather sparsely, with six macrosetae. Sternite VIII (Fig. 23): basal suture fully developed; posterior margin rounded; ventral surface covered with setae rather sparsely, with approximately nine macrosetae. Spermatheca (Fig. 26): deformed M-shaped; spermathecal head curved at middle; attachment of spermathecal duct inconspicuous; basal part of spermathecal stem moderate in size, clearly longer than spermathecal neck, with approximately ten coils attached complicatedly at base; each part of spermatheca entirely and very moderately sclerotized; inner wall of spermathecal head and neck, along border with head, finely and densely striate irregularly.

##### Etymology.

The replacement name is derived from “Eos” of the Greek mythology which is a Titaness and the goddess of the dawn because “Nippon” (= Japan, type locality) means a country of the dawn.

##### Distribution.

Japan, Taiwan (new record).

##### Bionomics.

One individual was caught with a flight interception trap (FIT).

##### Host records.

No host record is available.

##### Remarks on type materials.

Five syntypes were found. Among them, a male specimen (Fig. 35) labeled “Tinotus / japonicus / TYPE Cam [HW]” is designated as the lectotype herein.

##### Comments.

Since the name *Aleochara japonica* was already preoccupied by Sharp (1874), a new replacement name, Aleochara (Tinotus) eoa nom. n., for *Tinotus japonicus* Cameron, 1933 [nec. Sharp, 1874: 8 (*Aleochara*)], is proposed herein. No record of this species exists since its original description.

#### Aleochara (Tinotus) takashii sp. n.

Taxon classificationAnimaliaColeopteraStaphylinidae

http://zoobank.org/79D2ADA2-E87B-4F16-867C-904F9A438C20

Figs 3 , 27–34

##### Type locality.

Japan, Honshû: Takahachiyama, Fujinomiya City, Shizuoka Prefecture.

##### Type material.

Holotype: male, “Takahachiyama / Fujinomiya-shi / Shizuoka, JAPAN / 17-24. VIII. 2010 / T. Watanabe leg. [printed] // Flight / Intercept. / Trap [printed] // Aleocharini / Gen. / sp. / det. T. Watanabe 2013 [yellow square paper card, printed]” (KUM).

Paratypes: 1 male, “Teppogino-atama / Nishitanzawa / Kanagawa, Japan / 5-12. VII. 2007 / T. Watanabe leg. // Flight / Intercept. / Trap” (KUM); 1 male, “Teppogino-atama / Nishitanzawa / Kanagawa, Japan / 5-12. VII. 2007 / T. Watanabe leg. // Flight / Intercept. / Trap // *Aleochara* / sp. / det. T. Watanabe 2007” (KUM); 1 male, “Teppogino-atama / Nishitanzawa / Kanagawa, Japan / 5-12. VII. 2007 / T. Watanabe leg. // Flight / Intercept. / Trap // *Aleochara* / sp. / det. T. Watanabe 2008” (KUM); 1 male, “Idenzawa / Nishitanzawa / Kanagawa, Japan / 31. V – 6. VI. 2006 / T. Watanabe leg. // *Aleochara* / sp. / det. T. Watanabe 2007” (KUM); 2 spec., “Yanagisawa-toge / Enzan-shi / Yamanashi, Japan / 2-9. VIII. 2006 / T. Watanabe leg. // Flight / Intercept. / Trap // *Aleochara* / sp. / det. T. Watanabe 2007” (KUM); 1 female, “Yanagisawa-toge / Enzan-shi / Yamanashi, Japan / 9-15. VIII. 2006 / T. Watanabe leg. // Flight / Intercept. / Trap // *Aleochara* / sp. / det. T. Watanabe 2006” (PCTW); 2 females, “Karumizu-rindo / Narusawa-mura / Yamanashi, JAPAN / 30. VIII-14. IX. 2010 / T. Watanabe leg. // *Aleochara* / sp. / det. T. Watanabe 2012” (KUM); 1 female, “Karumizu-rindo / 1600 m, Narusawa / Yamanashi, JAPAN / 3-10. VIII. 2011 / T. Watanabe leg. // Flight / Intercept / Trap // *Aleochara* / sp. / det. T. Watanabe 2012” (KUM); 1 male, “Aokigahara, Fuji- / Kawaguchiko / Yamanashi, JAPAN 11-17. V. 2012 / T. Watanabe leg. // Flight / Intercept. / Trap / *Aleochara* / sp. / det. T. Watanabe 2013” (KUM); 1 male (head mounted on slide), 1 spec., “Fujisan 1-gome / Subashiri (1400 m) / Shizuoka, JAPAN / 20-26. V. 2011 / T. Watanabe leg. // Flight / Intercept. Trap // *Aleochara* / sp. / det. T. Watanabe 2012” (KUM); 1 male, 1 female, “Ohbuchi (alt. 950m) / Fuji-shi / Shizuoka, JAPAN / 13-18. V. 2010 / T. Watanabe leg. // Flight / Intercept. / Trap // *Aleochara* / sp. / det. T. Watanabe 2012” (KUM); 1 spec., “Ohbuchi (alt. 950m) / Fuji-shi / Shizuoka, JAPAN / 16-22. VII. 2010 / T. Watanabe leg. // Flight / Intercept. / Trap // *Aleochara* / sp. / det. T. Watanabe 2012” (KUM); 1 spec., “Ohbuchi (alt. 950m) / Fuji-shi / Shizuoka, JAPAN / 24. X. 2012 / T. Watanabe leg. // Aleocharinae” (KUM); 1 male, “Takahachiyama / Fujinomiya-shi / Shizuoka. JAPAN / 28. VIII. 2012 / T. Watanabe leg. // Aleocharinae” (KUM); 1 male, “Nishiusuzuka / Fujinomiya-shi / Shizuoka, JAPAN / 22-31. V. 2013 / T. Watanabe leg. // Flight / Intercept. / Trap // *Aleochara* / sp. / det. T. Watanabe 2013” (PCTW); 1 male, “Nishiusuzuka / Fujinomiya-shi / Shizuoka, JAPAN / 8-16. VII. 2010 / T. Watanabe leg. // Flight / Intercept. / Trap // *Aleochara* / sp. / det. T. Watanabe 2010” (PCTW); 1 male, 1 spec., “Nishiusuzuka / Fujinomiya-shi / Shizuoka, JAPAN / 16-22. VII. 2010 / T. Watanabe leg. // Flight / Intercept. / Trap // *Aleochara* / sp. / det. T. Watanabe 2011” (KUM); 1 spec., “Nishiusuzuka / Fujinomiya-shi / Shizuoka, JAPAN / 17-24. VIII. 2010 / T. Watanabe leg. // Flight / Intercept. / Trap // *Aleochara* / sp. / det. T. Watanabe 2012” (KUM).

##### Diagnosis.

**Figures 27–34.**
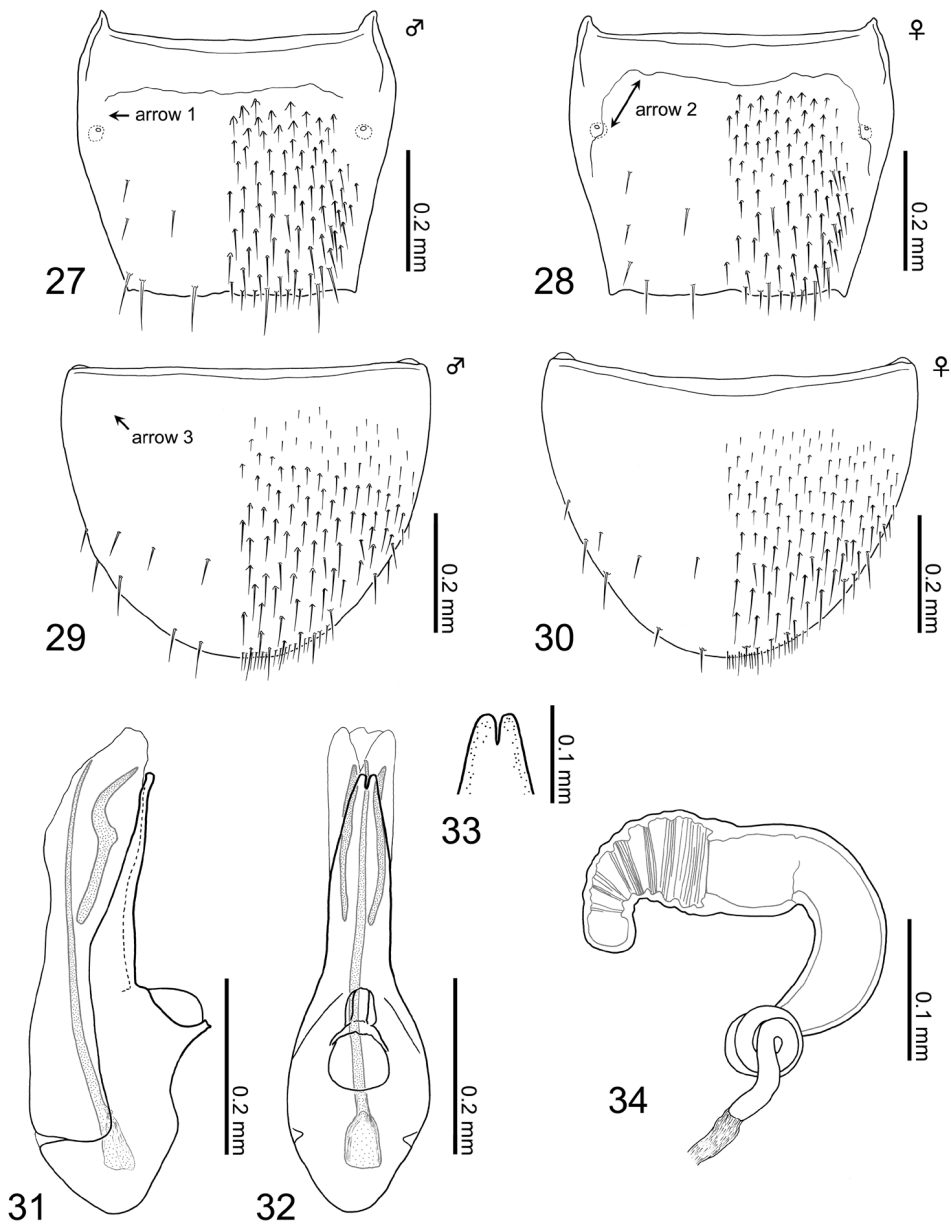
Terminalia of Aleochara (Tinotus) takashii: **27** tergite VIII of male **28** tergite VIII of female **29** sternite VIII of male **30** sternite VIII of female **31** median lobe of male aedeagus, lateral view **32** ditto, parameral view **33** ditto, apex of apical lobe, parameral view **34** female spermatheca.

This species can be easily distinguished from the other members of the subgenus by a following combination of characters: body entirely reddish brown (Fig. 3); abdominal segments III-V (first three visible terga) deeply impressed laterobasally; both tergite and sternite VIII with weakly developed basal sutures (Figs 27–30); median lobe of aedeagus with long apical lobe, notched deeply and medially at apex in ventral view (Fig. 33); spermatheca with a curved spermathecal head, unequally serrated inner walls inside spermathecal head, and with approximately three coils at base (Fig. 34). *Aleochara takashii* is the most similar to the North American species, Aleochara (Tinotus) imbricata (Casey, 1894), comb. n., of which shares including the similar configuration of male genitalia. From *Aleochara imbricata*, it can be distinguished additionally by having much more developed sclerites inside the median lobe of the aedeagus and by overall shape of the spermatheca (Klimaszewski et al. 2002: 289: Figs 13–16, 31–33).

**Figures 35–36.**
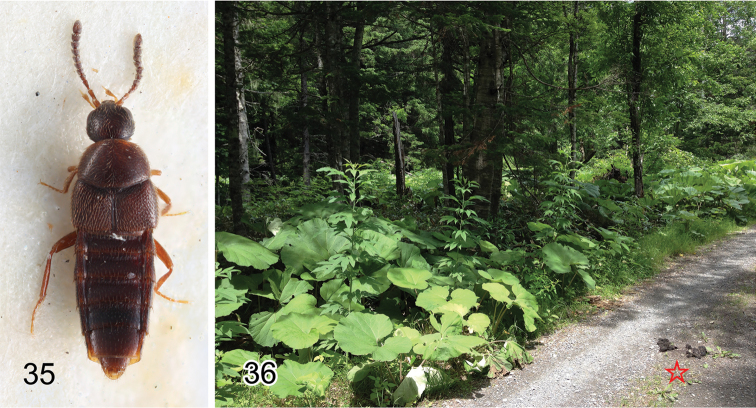
Type material and habitat of Aleochara (Tinotus): **35** lectotype of *Tinotus japonicus* (= *Aleochara eoa*) **36** Habitat of Aleochara (Tinotus) morion at Shihoro (Hokkaidô, Japan), red star indicating bear dung where eight specimens were collected.

##### Description.

*Measurements* (in mm, n = 23): BL = 3.156 (2.637–3.669); HL = 0.419 (0.361–0.483); HW = 0.488 (0.418–0.760); PL = 0.528 (0.421–0.605); PW = 0.756 (0.599–0.866); EW = 0.880 (0.693–1.019).

*Body* (Fig. 3): fusiform, compact, and robust; dorsal surface moderately glossy and pubescent, covered with small and inconspicuous micro-reticulation.

*Color* (Fig. 3): usually uniformly dark reddish brown to dark brown; elytra lighter; antennomeres I–IV lighter, but segments V to XI darker with numerous minute whitish setae; mouthparts and legs yellowish brown to dark reddish brown; apices of tergites III-V pale reddish brown transversely; pubescence yellowish brown to brown.

*Head* (Fig. 3): subquadrate, slightly transverse (HW/HL = 1.17, n = 23), widest just behind base of eyes; setae on vertex rather dense, directed anteriomedially. Eyes: small, occupying approximately one third of head length, very slightly protruding laterally.

*Antennae* (Fig. 3): short, moderately shorter than head and pronotum combined; relatively thick, setaceous, becoming gradually and moderately broaden apically in segments V to X, with segment V elongate and segments VI to X clearly transverse; segment XI symmetrical, obtusely pointed at apex; approximate relative length of segments from basal to apex: 23: 16: 17: 10: 9: 9: 9: 9: 9: 9: 22.

*Pronotum* (Fig. 3): strongly convex above dorsally, transverse (PW/PL = 1.43, n = 23), moderately longer than sutural length of elytra, widest around below of basal half, basal margin weakly rounded; pubescence in moderate length but thin, directed laterally and posterolaterally; micro-reticulation inconspicuous.

*Elytra* (Fig. 3): together, transverse, rather small, widest at middle; pubescence short, finely scattered densely, diverging posterolaterally in each elytron; dorsal surface moderately rough, shallowly impressed; posterolateral corner of each elytron moderately sinuate.

*Abdomen* (Fig. 3): first three visible tergites deeply impressed transversely at base; dorsal and ventral surface covered with setae rather sparsely.

*Male*. Tergite VIII (Fig. 27): basal suture not fully developed, suture partially disappeared laterally (Fig. 27: arrow 1); posterior margin very weakly serrate, insignificantly emarginate medially or truncate; dorsal surface covered with setae rather densely, with six macrosetae. Sternite VIII (Fig. 29): basal suture completely lost (Fig. 29: arrow 3); posterior margin rounded; ventral surface covered with short setae densely, with approximately eight macrosetae. Median lobe of aedeagus (Figs 31–33): narrowly elongate in lateral, and limuloid narrowly in parameral view; apical lobe slender and long, as long as basal capsule, moderately narrowing apically in lateral view, with deeply notched apex medially in parameral view (Fig. 33); without a protuberance at base of apical lobe; a pair of sclerites curved just above middle, long, approximately 2/3 length of apical lobe; flagellum developed, shorter than median lobe, without any coils at base.

*Female*. Tergite VIII (Fig. 28): basal suture not fully developed, suture partially disappeared laterally like male (see Fig. 27: arrow 1) or at most weakly developed (Fig. 28: arrow 2); posterior margin very weakly serrate or almost truncate; dorsal surface covered with setae densely, with six macrosetae. Sternite VIII (Fig. 30): basal suture completely lost like male (see Fig. 29: arrow 3); posterior margin rounded; ventral surface covered with setae densely, with approximately eight macrosetae. Spermatheca (Fig. 34): curved semi-circularly in lateral view; spermathecal head curved at middle; attachment of spermathecal duct inconspicuous; basal part of spermathecal stem moderate in size, clearly longer than spermathecal neck, with approximately three coils; each part of spermatheca entirely and very moderately sclerotized; inner wall of spermathecal head, coarsely striate irregularly.

##### Etymology.

The species name is dedicated to its collector, Mr. Takashi Watanabe (Kanagawa, Japan).

##### Distribution.

Only known from central Honshû, Japan (Kanagawa, Yamanashi, and Shizuoka Prefectures).

##### Bionomics.

Most specimens were caught with flight interception traps (FIT).

##### Host records.

No host record is available.

##### Remarks.

This new species is distinct among the species of *Tinotus*. In particular, reduced or non-developed basal sutures on tergite and sternite VIII of both sexes are notable character states (Figs 27–30). Furthermore, the apical lobe of the median lobe of male aedeagus, [i.e., deeply notched medially at apex in parameral view (Fig. 33)], is also a remarkable character state even among the subfamily. Since other morphological characters correspond fully to that of the subgenus *Tinotus*, we assign this species to *Tinotus* without hesitation.

### Key to species of the subgenus *Tinotus* (genus *Aleochara*) from Japan, Taiwan, and the Russian Far East

**Table:** 

1	Body black to blackish brown including pronotum and elytra (Fig. 1); median lobe of aedeagus of male with a basal protuberance in lateral view (Fig. 16); spermatheca coiled four times basally, with oblong and simple spermathecal head (Fig. 19)	**Aleochara (Tinotus) morion Gravenhorst, 1802, comb. n.**
–	Body dark brown to reddish brown (Figs 2, 3); median lobe of aedeagus of male without a basal protuberance in lateral view (Figs 24, 31); spermatheca with curved and deformed spermathecal head (Figs 26, 34)	**2**
2	Abdominal segments III-V (first three visible terga terga) deeply impressed laterobasally; tergite and sternite VIII with reduced basal sutures (Figs 27–30); median lobe of male aedeagus with short flagellum (Figs 31–32); spermatheca with approximately three coils at base (Fig. 34)	**Aleochara (Tinotus) takashii sp. n.**
–	Abdominal segments III-V (first three visible terga terga) rather shallowly impressed laterobasally; tergite and sternite VIII with complete basal sutures (Figs 20–23); median lobe of male aedeagus with long and coiled flagellum (Figs 24, 25); spermatheca with complex multiple coils at base (Fig. 26)	**Aleochara (Tinotus) eoa nom. n.**

## Discussion

We recognize three *Tinotus* species from Japan. This species count is clearly lower than those for mainland China (6 spp.: Pace 2013a; Schülke and Smetana 2015) and the United States (6 spp.: Klimaszewski et al. 2002; Gusarov 2003). In contrast, it surpasses the counts of adjacent countries, e.g., South and North Korea (0 sp.: Schülke and Smetana 2015) and Russia, including the Far East (1 sp.; Schülke and Smetana 2015; present study). Surprisingly, all of the continental European countries has no, or at most, only one *Tinotus* species (*Aleochara morion*; Schülke and Smetana 2015). Within central Honshû, Japan, we found all of the three species. Therefore, Japan, especially central part of the country, is considered to possess a relatively high species diversity of *Tinotus*.

Only one species has been confirmed in Taiwan and the Russian Far East, respectively, which may reflect potentially low species diversity in these regions or merely insufficient accumulation of materials. Since taxonomic studies and records of *Tinotus* in East Asia, including these two regions, are still lacking, further discussions are avoided here. The finding of *Aleochara eoa* in Taiwan implies a wide distributional range of this species, and it may be discovered on mainland China.

## Supplementary Material

XML Treatment for Tinotus

XML Treatment for Aleochara (Tinotus) morion

XML Treatment for Aleochara (Tinotus) eoa

XML Treatment for Aleochara (Tinotus) takashii

## Appendix

Checklist of the world species of the subgenus *Tinotus* of the genus *Aleochara*. Forty species are recognized in total. See annotated checklist of world species of *Tinotus* by Hanley (2002) for further details.

### Genus *Aleochara* Gravenhorst, 1802

#### Subgenus *Tinotus* Sharp, 1883: 170, stat. n.

1. *acerba* (Casey, 1911: 15), **comb. n.** (*Acrimea*). Distribution: Canada, USA.

= *resecta* (Casey, 1911: 14) (*Acrimea*).

2. *andensis* (Pace, 2008a: 365), **comb. n.** (*Tinotus*). Distribution: Ecuador.

3. *arawakorum* (Pace, 1990: 74), **comb. n.** (*Tinotus*). Distribution: Brazil (São Paulo).

4. *boreoindica* **nom. n.** [for *indica* (Cameron, 1939: 557) (*Tinotus*), nec. Fauvel, 1904: 66 (*Maseochara*), nec. Cameron, 1939: 635 (*Aleochara*)]. Distribution: India, China (Hubei, Shaanxi).

Etymology. Meaning northern India where the type locality is located.

5. *caelatimas* (Pace, 2008b: 177), **comb. n.** (*Tinotus*). Distribution: Malaysia (Sabah).

6. *carnivora* (Cameron, 1920: 270), **comb. n.** (*Paratheta*), nec. Gravenhorst, 1806: 171 (*Aleochara*). Distribution: Singapore.

7. *caviceps* (Casey, 1894: 316), **comb. n.** (*Tinotus*). Distribution: Canada, USA.

= *parata* (Casey, 1911: 64) (*Tinotus*).

8. *cavicollis* (Sharp, 1883: 170), **comb. n.** (*Tinotus*). Distribution: USA (FL), Guatemala, Nicaragua, Brazil, Paraguay, Argentina, Cuba.

9. *clavicornuta* **nom. n.** [for *clavicornis* (Cameron, 1945: 729) (*Tinotus*), nec. L. Redtenbacher, 1849: 822 (*Aleochara*)]. Distribution: South Africa.

Etymology. Small change of the older specific epithet for this species, *clavicornis*, meaning clavate antenna.

10. *densula* Wasmann, 1900: 240, **comb. n.** Distribution: South Africa.

11. *eoa* **nom. n.** [for *japonica* (Cameron, 1933: 217) (*Tinotus*), nec. Sharp, 1874: 8 (*Aleochara*)]. Distribution: Japan, Taiwan (new record).

Etymology. See, the redescription section above.

12. *flavescens* (Sharp, 1883: 171), **comb. n.** (*Tinotus*). Distribution: Mexico, Guatemala.

13. *frontalis* (Pace, 1997: 45), **comb. n.** (*Tinotus*), nec. Stephens, 1832: 111 (*Aleochara*). Distribution: Colombia.

14. *globicollis* (Bernhauer, 1934b: 19), **comb. n.** (*Tinotus*). Distribution: China (Sichuan), Philippines.

15. *imbricata* (Casey, 1894: 317), **comb. n.** (*Tinotus*). Distribution: USA.

= *texana* (Casey, 1911: 67) (*Tinotus*), nec. Casey, 1906: 137 (*Aleochara*).

= *coelebs* (Casey, 1911: 68) (*Tinotus*).

= *fusina* (Casey, 1911: 68) (*Tinotus*).

= *pectinella* (Casey, 1911: 69) (*Tinotus*).

= *ampla* (Notman, 1920: 723) (*Tinotus*).

= *brunnipes* (Notman, 1920: 724) (*Tinotus*), nec. Stephens, 1832: 133 (*Aleochara*).

16. *janklimaszewskii* **nom. n.** [for *klimaszewskii* (Pace, 2008a: 365) (*Tinotus*), nec. Maus, 1999: 358 (*Aleochara*)]. Distribution: Ecuador.

Etymology. Dedicated to Dr. Jan Klimaszewski, as the older specific epithet for this species.

17. *kashmirica* (Cameron, 1939: 559), **comb. n.** (*Tinotus*). Distribution: India (Kashmir, Himachal Pradesh).

18. *malaya* **nom. n.** [for *antennalis* (Cameron, 1950a: 128) (*Tinotus*), nec. Fenyes 1914: 54 (*Aleochara*)]. Distribution: Malaysia (Malay Peninsula).

Etymology. An old name of Peninsular Malaysia where the type locality is located.

19. *morion* Gravenhorst, 1802: 97, **comb. n.** Distribution: Holarctic region (native to Palaearctic region).

= *exigua* Mannerheim, 1930: 68 (*Aleochara*).

20. *namibiensis* (Pace, 1999b: 207), **comb. n.** (*Tinotus*). Distribution: Namibia.

21. *natalensis* (Pace, 1986: 107), **comb. n.** (*Tinotus*). Distribution: Kenya, Zimbabwe, South Africa.

22. *ndogo* **nom. n.** [for *minuta* (Bernhauer, 1915b: 158) (*Tinotus*), nec. Casey, 1906: 161 (*Baryodma*)]. Distribution: Zaire, Tanzania, Kenya, South Africa.

= *suffusa* (Cameron, 1950b: 76) (*Tinotus*), nec. Casey, 1906: 162 (*Baryodma*).

23. *neocaledonica* (Pace, 1991: 165), **comb. n.** (*Tinotus*). Distribution: New Caledonia.

Etymology. Swahili adjective ndogo meaning small in referring to the minute body of this species.

24. *nepalensis* (Pace, 1984: 338), **comb. n.** (*Tinotus*). Distribution: Nepal.

25. *olivosensis* **nom. n.** [for *densissima* (Bernhauer, 1934a: 512) (*Tinotus*), nec. Bernhauer, 1906: 345 (*Aleochara*)]. Distribution: Brazil (Rio de Janeiro), Argentina.

Etymology. Derived from the type locality of *densissima*, Olivos, Buenos Aires of Argentina.

26. *papuana* (Pace, 2000: 161), **comb. n.** (*Tinotus*). Distribution: Papua New Guinea.

27. *planula* (Notman, 1920: 724), **sp. rev., comb. n.** (*Tinotus*). Distribution: USA.

= *parvicornis* (Casey, 1911: 69), **syn. n.** (*Tinotus*), nec. Fauvel, 1900: 248 (*Aleochara*).

= *densiventris* (Casey, 1911: 70) (*Tinotus*), nec. Bernhauer, 1906: 346 (*Aleochara*), nec. Casey, 1906: 158 (*Baryodma*).

28. *refusa* (Hanley, 2002: 454), **comb. n.** (*Tinotus*). Distribution: Mexico.

29. *riodejaneirensis* **nom. n.** [for *eidmanni* (Scheerpeltz, 1936: 528) (*Tinotus*), nec. Scheerpeltz, 1929: 137 (*Aleochara*)]. Distribution: Brazil (Rio de Janeiro).

Etymology. Derived from Rio de Janeiro, the capital city of Brazil and the type locality of this species.

30. *robertopacei* **nom. n.** [for *major* (Pace, 1986: 107) (*Tinotus*), nec. Fairmaire, 1858: 737 (*Aleochara*), nec. Eichelbaum, 1912: 176 (*Aleochara*)]. Distribution: South Africa, Tanzania, Kenya.

Etymology. Dedicated to Mr. Roberto Pace who first described this species.

31. *rougemonti* (Pace, 1993: 116), **comb. n.** (*Tinotus*). Distribution: China (Sichuan, Xinjiang).

32. *rougemontiana* (Pace, 1999a: 152), **comb. n.** (*Tinotus*). Distribution: China (Shaanxi, Sichuan, Yunnan).

33. *surrubicunda* **nom. n.** [for *rufipennis* (Cameron, 1939: 558) (*Tinotus*), nec. Stephens, 1832: 161 (*Aleochara*), nec. Erichson, 1839: 162 (*Aleochara*)]. Distribution: India (Uttar Pradesh, Uttarranchal), Nepal.

Etymology. Combination of the Latin prefix *sur* meaning above or upper and the Latin adjective *rubicunda* meaning red in referring to the reddish body of this species.

34. *takashii* **sp. n.** Distribution: Japan (central Honshû).

35. *taprobanensis* (Likovský, 1984: 4), **comb. n.** (*Tinotus*). [replacement name for *Aleochara minutissima* Kraatz, 1859: 19]. Distribution: Sri Lanka, Malaysia (Penang), Himalaya.

= *minutissima* Kraatz, 1859: 19 (*Aleochara*).

36. *thailandensis* (Pace, 1992: 260), **comb. n.** (*Tinotus*). Distribution: Thailand.

37. *trisecta* (Casey, 1906: 321), **comb. n.** (*Tinotus*). Distribution: Canada, USA.

= *fimbriata* (Casey, 1911: 15) (*Acrimea*).

= *pallida* (Casey, 1911: 65) (*Tinotus*).

= *brunnea* (Casey, 1911: 65) (*Tinotus*), nec. Motschulsky, 1860: 582 (*Caladera*).

= *binaria* (Casey, 1911: 66) (*Tinotus*).

= *lateralis* (Notman, 1821: 154) (*Tinotus*).

38. *uttariana* **nom. n.** [for *castanea* (Cameron, 1939: 559) (*Tinotus*), nec. Motschulsky, 1858: 239 (*Aleochara*)]. Distribution: India (Uttar Pradesh), Nepal, China (Sichuan).

Etymology. Derived from the type locality of *castanea*, Uttar Pradesh, India.

39. *vietnamensis* (Pace, 2001: 143), **comb. n.** (*Tinotus*). Distribution: Vietnam.

40. *zairensis* (Pace, 1986: 107), **comb. n.** (*Tinotus*). Distribution: Zaire.

### Genus *Aleochara* Gravenhorst, 1802

#### Subgenus *Aleochara* Gravenhorst, 1802: 67.

1. *derougemonti* **nom. n.** [for *rougemonti* Pace, 2011: 68 (*Aleochara*), nec. Pace, 1993: 116 (*Tinotus*)]. Distribution: Venezuela.

Etymology. Dedicated to Mr. Guillaume de. Rougemont as the older specific epithet for this species.

2. *vietnamiana* **nom. n.** [for *vietnamensis* Pace, 2013b: 376 (*Aleochara*), nec. Pace, 2001: 143 (*Tinotus*)]. Distribution: Vietnam.

Etymology. Small change of the he older specific epithet for this species, meaning “of Vietnam”.

#### Subgenus *Xenochara* Mulsant & Rey, 1874: 60.

1. *natalicola* **nom. n.** [for *natalensis* Klimaszewski, 1993: 67 (*Aleochara*), nec. Pace, 1986: 107 (*Tinotus*)]. Distribution: South Africa.

Etymology. Combination of Natal, the type locality of this species, and the Latin suffix *cola* meaming inhabitor.
